# Revolutionizing Cancer Care: Advances in Carbon-Based Materials for Diagnosis and Treatment

**DOI:** 10.7759/cureus.52511

**Published:** 2024-01-18

**Authors:** Muhammad Zubair Khan, Danial Tahir, Muhammad Asim, Muhammad Israr, Ali Haider, Dan Dan Xu

**Affiliations:** 1 Internal Medicine, Ayub Teaching Hospital, Abbottabad, PAK; 2 Internal Medicine, Nazareth Hospital, Philadelphia, USA; 3 Internal Medicine, Royal Infirmary of Edinburgh, NHS Lothian, Edinburgh, GBR; 4 Biological Sciences, Abasyn University, Islamabad, PAK; 5 Department of Allied Health Sciences, The University of Lahore, Gujrat Campus, Gujrat, PAK; 6 Integrative Medicine, Shandong University of Traditional Chinese Medicine, Jinan, CHN

**Keywords:** cancer care, carbon-based materials, chemotherapy, nanomaterials, cancer therapy, nanotechnology, cancer treatment, carbon quantum dots, cancer diagnosis

## Abstract

Cancer involves intricate pathological mechanisms marked by complexities such as cytotoxicity, drug resistance, stem cell proliferation, and inadequate specificity in current chemotherapy approaches. Cancer therapy has embraced diverse nanomaterials renowned for their unique magnetic, electrical, and optical properties to address these challenges. Despite the expanding corpus of knowledge in this area, there has been less advancement in approving nano drugs for use in clinical settings. Nanotechnology, and more especially the development of intelligent nanomaterials, has had a profound impact on cancer research and treatment in recent years. Due to their large surface area, nanoparticles can adeptly encapsulate diverse compounds.

Furthermore, the modification of nanoparticles is achievable through a broad spectrum of bio-based substrates, including DNA, aptamers, RNA, and antibodies. This functionalization substantially enhances their theranostic capabilities. Nanomaterials originating from biological sources outperform their conventionally created counterparts, offering advantages such as reduced toxicity, lower manufacturing costs, and enhanced efficiency. This review uses carbon nanomaterials, including graphene-based materials, carbon nanotubes (CNTs) based nanomaterials, and carbon quantum dots (CQDs), to give a complete overview of various methods used in cancer theranostics. We also discussed their advantages and limitations in cancer diagnosis and treatment settings. Carbon nanomaterials might significantly improve cancer theranostics and pave the way for fresh tumor diagnosis and treatment approaches. More study is needed to determine whether using nano-carriers for targeted medicine delivery may increase material utilization. More insight is required to explore the correlation between heightened cytotoxicity and retention resulting from increased permeability.

## Introduction and background

Despite notable advancements in cancer treatment, the limited repertoire of effective methods persists, with a paucity of understanding regarding the underlying processes of metastatic disease and cancer recurrence, leading to significant morbidity and mortality [1]. Mutations in DNA are the driving force behind cancer development. In 2022, it was anticipated that the United States would see approximately 1,918,030 new cancer cases and 609,360 deaths stemming from this condition. Lung cancer is the primary cause of daily cancer-related deaths, claiming nearly 10 million lives per year [2]. The Global Cancer Observatory projects approximately 30 million annual cancer-related deaths by 2030, imposing a substantial financial burden on families and society. Prioritizing cancer prevention, screening, diagnosis, and treatment is imperative [3]. Dysfunctional cell-cycle regulation and impaired apoptosis contribute to cancer cell proliferation and metastasis. Altered signaling pathways are associated with cancer development and resistance to treatment is linked to the failure of apoptosis under physiological conditions [4]. Like their association in cancer patients, inflammation and immune system malfunction are linked. Using the AJCC/UICC-TNM classification, traditional tumor staging considers tumor tissue quantity, malignant cells in draining lymph nodes, and tumor metastases [5]. Nanoparticles, ranging from 10 nm to 100 nm, offer a large surface area and are ideal for applications in the biological sciences. Their high mobility facilitates easy transport to various organs and effective penetration into targeted regions. Conjugates with therapeutic molecules enable selective drug delivery to diseased tissues, such as cancer cells, and their size is comparable to DNA but significantly smaller than red blood cells [6]. This enhances performance and imparts distinctive physical, chemical, and optical properties, valuable in the medical field for cancer treatment and detection. These nanoparticles can introduce novel approaches and enhance established methods.

**Figure 1:**
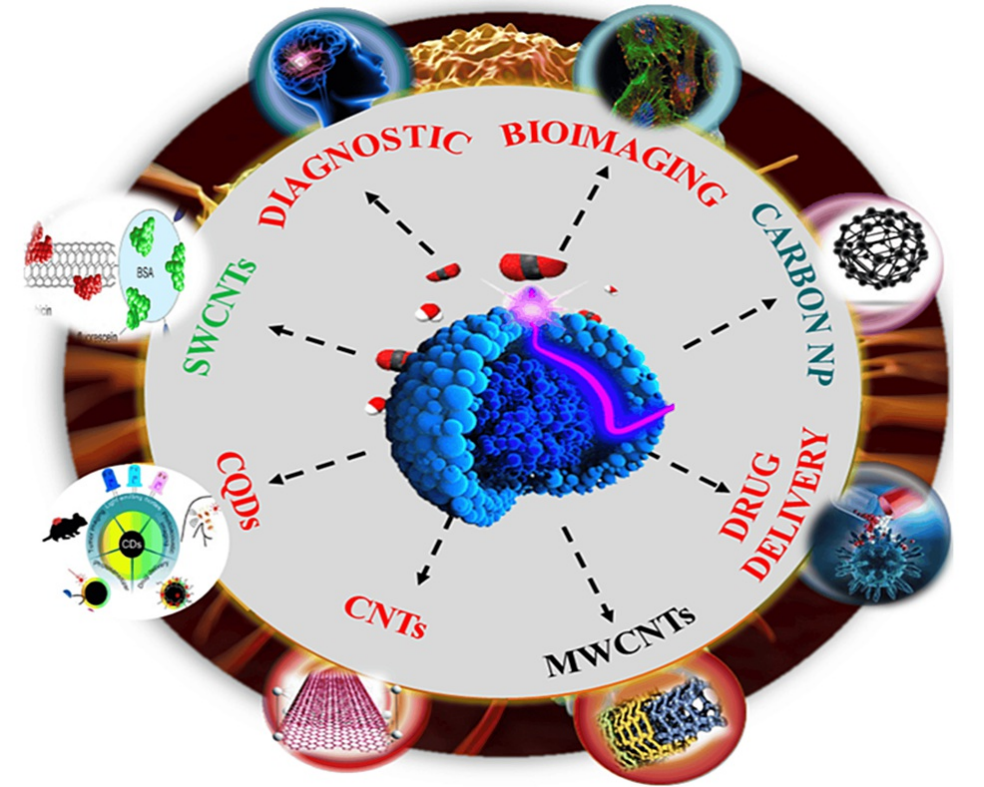
Applications of carbon-based materials for diagnosis and treatment of cancer CQDs: carbon quantum dots; CNTs: carbon nanotubes; MWCNTs: multi-walled carbon nanotubes; SWCNTs: single-walled carbon nanotubes; NP: nanoparticles Note: This image is the author's creation.

Nanoparticles, serving as targeting agents, enable precise localization of molecules within cancer cells, enhancing the efficiency of cancer imaging tools and ultimately improving detection. Recent research has extensively explored the capacity of nanoparticles to enhance cancer imaging for more effective detection [7]. Nanomaterials are synthesized for specific applications, particularly in the detection and diagnosis of cancer, with a primary goal of creating materials that are both effective and easily deliverable to cancerous tumor cells [8]. This review explored various types of nanoparticles, such as carbon nanotubes and graphene-based materials, utilized in cancer detection, imaging, and treatment due to their diverse biological applications. Their unique characteristics, including chemical, magnetic, and optical properties, have led to their utilization in various medical applications, enabling the development of diagnostic imaging probes and drug materials for cancer treatment. The progress in sophisticated nanomaterials presents a promising avenue for early cancer detection, diagnosis, and treatment. The review concludes by suggesting potential research gaps for future studies in this evolving field (Figure 1).

## Review

Nanoparticles: A game changer in cancer diagnosis

Various nanoparticles play a crucial role in cancer detection, including magnetic nanoparticles, quantum dots, polymeric nanoparticles, metallic nanoparticles, fullerene nanoparticles, liposomes, graphene nanotubes, and dendrimers. They contribute significantly to the imaging process, traversing biological barriers and circulating before reaching their destinations. Enhancements involve conjugating cancer-specific antibodies to nanoparticles for improved tumor binding and detection [7]. Recent studies suggest nanoparticles and sensors can enhance cancer diagnostics and detection sensitivity. Diagnostic indicators include methylation patterns and mutation identification, while clinical diagnosis methods using circulating tumor cells, cell-free RNA, and extracellular vesicles require further investigation [7]. Stabilized by trimethoxysilane, (3-Mercaptopropyl) fluorescent gold nanoclusters and superparamagnetic ferrite are incorporated with polyethylene glycol dimethacrylate to create Fe_3_O_4_/GNCs nanoprobes on "Fe_3_O_4_@SiO_2_ nanoparticles" [9]. Cancer-targeting antibodies exhibit enhanced infiltration into tumor cells with increased sensitivity when delivered via metallic nanoparticles, such as platinum, copper, silver, gold, and cobalt nanocomposites. These nanocomposites have demonstrated potential in cancer diagnostics and imaging, facilitating precise colon and breast cancer cell penetration and improved diagnostic signals. Because of their unique magnetic, chemical, and optical properties, nanoscale materials are employed to create imaging probes for positron emission tomography (PET), MRI, single-photon emission computed tomography (SPECT), and ultrasound, offering enhanced sensitivity, temporal and spatial information, and improved control over biodistribution, contrast, functionality, and multimodal imaging. These advancements promise future clinical benefits, including real-time disease monitoring, personalized therapy, and early diagnosis.

Nanoparticles as diagnostic imaging agents

Medical imaging plays a crucial role in diagnosing and treating malignant tumors. Nanoparticles, such as iron oxide NPs, leverage their optical, magnetic, acoustic, and structural features to advance imaging technologies. Research indicates that introducing nanoparticles into specific tissues enhances image contrast, aiding tumor diagnosis and surgical operations [9]. In cryosurgery, nanoparticles improve the imaging of both tumors and the ice ball border, resulting in clearer pictures that enable more effective therapy. Metallic nanoparticles are commonly used in various imaging applications, with the choice of metal dependent on the imaging principle employed [10]. Overall, nanoparticles, derived from diverse materials, are integral to enhancing clarity and efficacy in medical imaging diagnostics.

Optical coherence tomography (OCT) is a non-invasive imaging technique offering micron-level resolution within the biological sciences. Its significance in medical imaging is well-established, especially in real-time diagnostics and surgical guidance. Notably, OCT faces challenges in sensitivity to inelastically dispersed light due to the lack of coherence in the incident area [11]. According to recent studies, there is a promising way to overcome the limitations of OCT sensitivity. Researchers have found that the mobility of nanoparticles can affect the amplitude of OCT. This discovery suggests that controlling the mobility of nanoparticles could be a potential solution to the sensitivity issue in OCT. According to recent studies, there is a promising way to overcome the limitations of OCT sensitivity. Researchers have found that the mobility of nanoparticles can affect the amplitude of OCT. This discovery suggests that controlling the mobility of nanoparticles could be a potential solution to the sensitivity issue in OCT [12].

MRI is commonly used as the most reliable non-invasive method for detecting tumors. However, the accuracy of clinical detection of tumors is hindered by the challenge of comparing MRI signals between healthy and cancerous tissues [13]. MRI is a non-invasive technique that uses the magnetic properties of hydrogen nuclei in water molecules to reveal various characteristics of organs and tissues. Contrast agents can be used to enhance the clarity of images [14]. Contrast agents, especially magnetic nanoparticles, can exploit the enhanced permeability and retention effect associated with tumors. Iron oxide magnetic nanoparticles are preferred as MR imaging contrast agents due to their specificity in targeting specific cells [15].

For instance, they can be used to study the intracellular absorption of iron oxide nanoparticles by Kupffer cells in the liver to detect liver cancer. These nanoparticles exhibit low signal intensity in healthy tissue and high intensity in tumor tissue [16]. Recent research suggests that surface modification techniques and the use of tumor-specific bio-oligomers can help localize nanoparticles within tumors. This approach holds promise for detecting tiny tumors in their earliest stages. Trials using human transferrin targeting with gold nanoparticles (AuNPs) have significantly improved brain tumor imaging efficacy [17]. Combining paramagnetic nanoparticle probes with anti-epidermal growth factor receptor monoclonal antibodies has enabled the detection of malignancies in their earliest and most treatable stages [18].

Carbon nanotubes

Scientists have been fascinated with carbon nanotubes since they gained popularity in the 1990s. These nanostructures are created by folding graphene. Many types of carbon nanotubes have been proposed, including single-walled, double-walled, and multi-walled nanotubes [19]. Due to their compact size and cylindrical shape, carbon nanotubes display a range of intriguing properties. Single-walled carbon nanotubes, in particular, have desirable properties that may make them superior to other common building materials [19]. Researchers are investigating the potentially far-reaching effects of carbon nanotubes in the fields of biology and medicine. Several studies have been conducted to demonstrate their effectiveness in these contexts [20]. Recently, a matrix made of various permutations of DNA and carbon nanotubes has been proven effective in detecting biomarkers associated with gynecologic malignancies [21]. The impact of biomarkers on the optical properties of carbon nanotubes has been investigated and proven to be sequence- and chirality-dependent. Therefore, it may be possible to detect a cancer fingerprint by monitoring the fluorescence spectra of several DNA-CNT hybrids. This study demonstrates the potential of functional carbon nanotube-based nanostructures for the early detection and effective treatment of several cancer types, including pancreatic and liver tumors [21].

CNTs for pancreatic cancer analysis and therapy

Pancreatic cancer, often abbreviated as PC in medical literature, is a devastating disease that primarily affects the pancreas. Unfortunately, studies show that individuals diagnosed with this type of cancer have a very low chance of survival. The disease may spread to other organs and infiltrate nearby tissues [22]. Most individuals with pancreatic cancer are discovered in the later stages of the illness since there are no clinical signs in the early stages. Surgical methods become less effective in later stages due to the increasing risk of metastasis [23,24]. Traditional diagnostic and staging techniques include endoscopic ultrasonography and computed tomography in conjunction with a fine-needle biopsy. However, interpreting CT images requires expertise and precision to ensure a successful endoscopic operation [25]. As a result, developing a novel approach to pancreatic cancer detection is necessary. In recent years, researchers have shown an increasing interest in using carbon nanotubes for their various theranostic properties to create novel nanomaterials for cancer research [26]; biomarkers like carbohydrate antigen 19-9 (CA19-9) have also shown potential in cancer diagnosis. A biosensor using MWCNTs embedded in microporous filter paper has been developed to detect CA19-9, which may accurately diagnose cancers of the pancreas, bile duct, liver, stomach, and colorectal tract [27].

CNTs for liver cancer detection and therapy

Liver cancer is considered to be the sixth most common type of cancer in humans. It is more prevalent in Asia and Africa than in Europe [28]. Among all liver cancer cases, around 75% are either hepatocellular carcinoma or malignant hepatoma. Liver transplantation or other surgical intervention is the most effective treatment for liver cancer in the early stages. However, it is ineffective once the tumor progresses [28]. Currently, the primary approach to treating this illness involves therapy. Still, its effectiveness is hampered by challenges such as low selectivity in targeting liver cancer cells, widespread medication resistance, and the occurrence of unpleasant side effects. In hepatocellular carcinoma therapy, significant reliance on anti-angiogenesis medications, particularly tyrosine kinase inhibitors, exists. Unfortunately, while these medications aim to eradicate cancer, they can also damage healthy cells, affecting natural cell proliferation in various body parts, including the bone marrow, digestive system, and hair follicles. The emergence of nanotechnology has ushered in a new era of diagnostic and therapeutic techniques utilizing nano-carriers, offering potential solutions to these challenges. Carbon nanotubes have been instrumental in developing cutting-edge diagnostic tools for detecting liver cancer. Numerous studies have demonstrated that carbon nanotubes' advantageous biochemistry and microstructure play a crucial role in facilitating the targeted delivery of anticancer drugs to specific cancer cells, addressing some of the limitations associated with conventional chemotherapy [29].

CNTs for prostate cancer detection and therapy

Researchers conducted a study that involved combining polyethyleneimine-functionalized multi-wall carbon nanotubes with paclitaxel, a first-line therapy for prostate cancer. Researchers utilized PSMA antibodies to coat the particles, which were subsequently targeted to prostate cancer cells. In the laboratory, fluorescent CNT composites were exposed in vitro to PSMA+ HCT-116 LNCaP prostate cancer cells, PSMA CaCo-2 colon cancer cells, and PSMA human peripheral monocytes and lymphocytes. The interaction between CNT-PTX and CNTs with all cell types was confirmed through fluorescence microscopy and flow cytometry, revealing a widespread association. Cytotoxicity analysis demonstrated that combining paclitaxel with carbon nanotubes was more effective than pure paclitaxel or carbon nanotubes alone when targeting prostate and colorectal cancer cells. This innovative approach holds promise for enhancing the efficacy of cancer treatment by leveraging the synergistic effects of nanotube-assisted drug delivery [29].

CNTs for breast cancer detection and therapy

**Figure 2:**
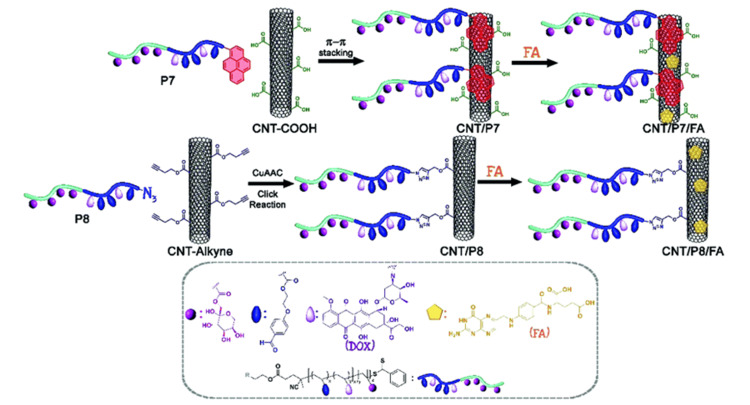
The schematic route illustrates the process of modifying carbon nanotubes (CNTs) using π−π stacking and CuAAC click reaction techniques CNT: carbon nanotubes, COOH: carboxylic acid, CNT-COOH: carboxylic acid-modified carbon nanotube, FA: folic acid, DOX: doxorubicin, P7: Dox-conjugated glycoblock copolymer P(FruMA-b-MAEBA)-Py/Dox, P8: Dox-conjugated glycoblock copolymer P(FruMA-b-MAEBA)-N3/Dox, CuAAC: Copper(I)-catalyzed azide-alkyne cycloaddition. Reproduced with permission from ref no: [31]. Copyright 2020 The Royal Society of Chemistry

Breast cancer is considered the most widespread type of cancer among women. It can be classified into subtypes based on molecular markers, which include hormone positive, Her2 negative or hormone negative, Her2 positive, or triple negative, i.e., negative ER, PR, and her2, etc. The primary molecular targets in breast cancer are Estrogen Receptor alpha, Transferrin Receptor, and Epidermal Growth Factor 2 [30]. Using a biocompatible copolymer, poly [1-O-methacryloyl--d-fructopyranose-b-(2-methacryloxyethoxy)] benzaldehyde glycoblock, researchers synthesised multifunctional carbon nanotubes. Synthesized functionalized glycoblock copolymers have doxorubicin attached to both ends (Figure 2). Two proteins, folate receptor and glucose transporter protein, have shown promise as therapeutic targets for breast cancer. They employed carbon nanotubes coated with folic acid and synthetic drug-conjugated glycoblock copolymers to create a very effective drug delivery method that targets both FR and GLUT5 [31].

**Figure 3:**
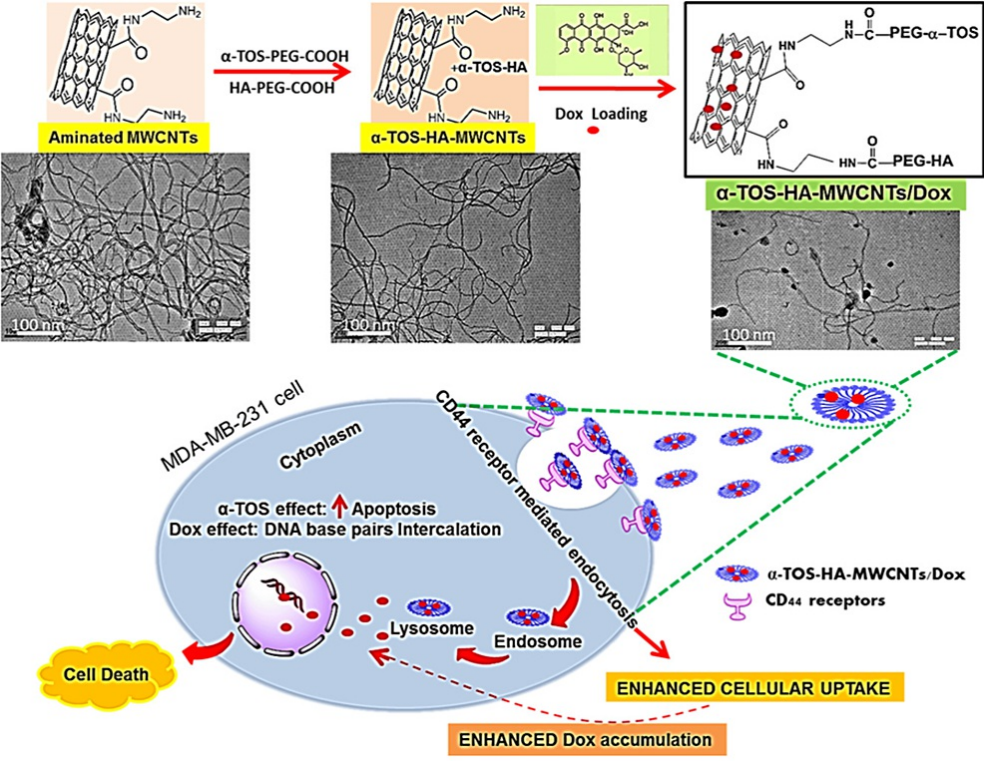
Mechanism of cell death by CD44 receptor mediated endocytosis by prepared OS-HA-MWCNTs/Dox MWCNTs: multi-walled carbon-nanotubes, HA: hyaluronic acid, α-TOS: α-tocopheryl succinate, Dox: doxorubicin, PEG: polyethylene glycol, COOH: carboxylic acid, α-TOS-PEG-COOH: α-tocopheryl succinate-polyethylene glycol-carboxylic acid, HA-PEG-COOH: hyaluronic acid-polyethylene glycol-carboxylic acid, α-TOS-HA-MWCNTs/Dox: novel conjugate obtained by functionalized MWCNTs using HA and α-TOS and loaded with Dox, MDA-MB-231: cell line (isolated at M D Anderson from a pleural effusion of a patient with invasive ductal carcinoma) is commonly used to model late-stage breast cancer. Reproduced with permission from ref. [32] Copyright 2020 Elsevier B.V.

The triple-negative breast cancer cell line MDA-MB-231 was shown to upregulate the CD44 receptor. Hyaluronic acid and alpha-tocopheryl succinate were studied by researchers for their potential as a targeted ligand for CD-44 receptors in breast cancer. The α-TOS-HA-MWCNTs/DOX conjugate facilitates targeted delivery of the conjugate to specific cells by chemically modifying the MWCNTs with hyaluronic acid and -tocopherol succinate in conjunction with doxorubicin. Notably, TOS-HA-MWCNTs/DOX showed preferable cellular absorption in comparison to other multi-wall carbon nanotube formulations that had been produced for this purpose. When functionalization of multi-wall carbon nanotubes was accomplished by HA, -TOS, and DOX.treated with MDA-MB-231 cells showed considerable growth inhibition and an elevated overall apoptotic ratio (Figure 3). The study concluded that HA, α-TOS might be employed for safe, synergistic tumor detection [32].

CNTs for colorectal cancer detection and therapy

According to recent epidemiological data, metastatic colorectal cancer has risen to the position of third most frequent cancer worldwide. The prognosis for colorectal cancer is good in the early stages but drops dramatically to 13% if stage IV (metastatic) disease has set in [33]. Due to the development of resistance to existing medications, they only marginally increase life expectancy. Metastatic colorectal cancer is one of the major causes of cancer-related death despite significant therapy advances over the last two decades. Overexpression of human epidermal growth factor receptor-2 is seen in around 3-5% of instances of metastatic colorectal cancer. The presence of HER2, a well-recognized biomarker with negative predictive value in metastatic colorectal cancer, decreases the effectiveness of chemotherapy targeting EGFR [33].

Researchers chemically altered SWCNTs using folic acid, capecitabine, and fluorescein, known anticancer agents commonly used in treating colorectal cancer. To take advantage of the synergistic interaction between the functionalized SWCNTs and the type-II nanocrystalline cellulose (II-NCC), they were suspended in water. The resultant colloidal system was tested in vitro on both normal (differentiated) and cancerous (proliferative) human colon cells. Functionalized SWCNT/II-NCC hybrids are more effective than the standard capecitabine in killing the Caco-2 cancer cell line [34]. To target colorectal cancer, Researchers synthesized gemcitabine-loaded hydroxyapatite conjugated multi-walled carbon nanotubes and tested their effectiveness in vitro and in vivo. In vitro and in vivo studies have been undertaken using aminated and PEGylated multi-walled carbon nanotubes and hyaluronic acid conjugate CNTs [35]. Release of GEM was shown to be more rapid under acidic circumstances (pH = 5.3) compared to physiological settings (PBS, pH = 7.4), followed by a progressive release pattern, as determined by in vitro tests. The GEM-loaded multifunctional MWCNTs were substantially less harmful to red blood cells and more hazardous to the HT-29 colon cancer cell line than the free GEM (18.71 0.44%). The anticancer investigation found that GEM/HA-PEG-MWCNTs dramatically decreased tumor volume compared to free GEM and increased survival rates without causing considerable weight loss. In tumor-bearing rats, the pharmacokinetic characteristics of multi-walled carbon nanotubes bonded with hydroxyapatite and loaded with gemcitabine and GEM-loaded multifunctional multi-walled carbon nanotubes significantly outperformed those of free GEM (p 0.001). Using modified multi-walled carbon nanotubes as nanomedicine for treating colorectal cancer is both effective and safe [35].

CNTs for brain cancer detection and therapy

**Figure 4:**
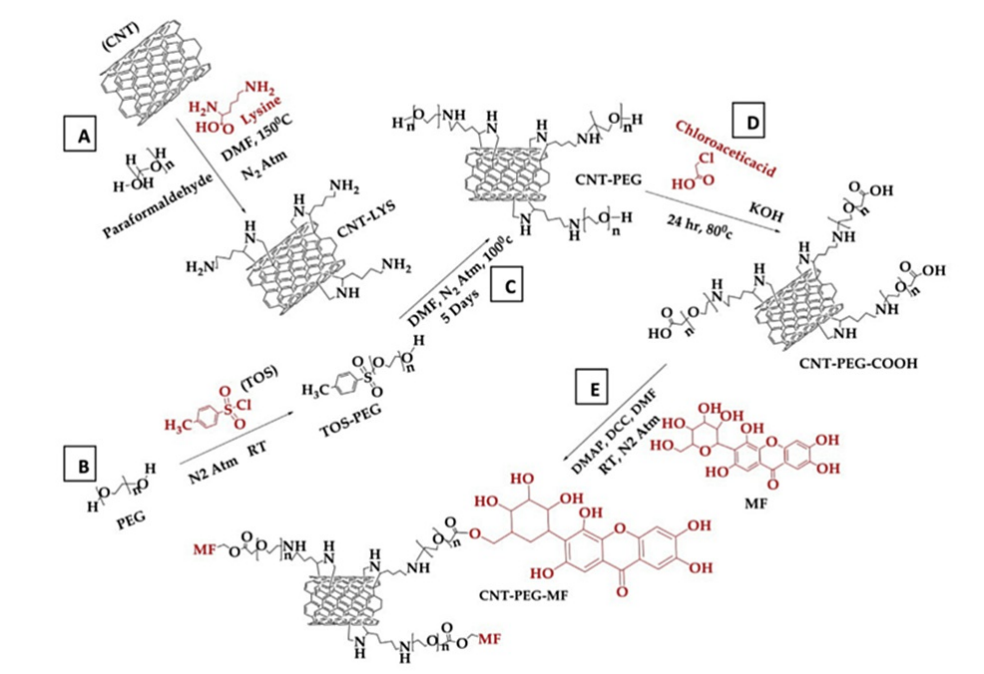
Diagram illustrating the process of creating a CNT-PEG-MF conjugate CNT: carbon nanotube, DMF: dimethylformamide, LYS: lysine, CNT-LYS:  lysine modified carbon nanotube, PEG: polyethylene glycol, COOH: carboxylic acid, CNT-PEG: polyethylene glycol modified carbon nanotubes, CNT-PEG-COOH: polyethylene glycol-carboxylic acid modified carbon nanotube, MF: Mangiferin, CNT-PEG-MF: polyethylene glycol-mangiferin modified carbon nanotube, TOS-PEG: tosylated polyethylene glycol. Reproduced with permission from ref. [38] Copyright 2019 Elsevier B.V.

The existence of the blood-brain barrier and the special need for drug physicochemical properties make formulation development for medicine delivery to the brain more challenging than for other organs. Therefore, nanoscale drug delivery may easily fulfill the needs for treating brain illnesses. Carbon nanotubes have been the subject of intense research in recent years due to their possible use in treating brain tumors. This is because, when paired with suitable ligands, they can efficiently transport various medicinal medicines [36]. Researchers employed PEGylated carbon nanotubes in an experiment with glioblastoma and astrocytoma cells. Nuclear magnetic resonance and Fourier transform infrared spectroscopy were used to examine the nanocomposites for particle size, zeta potential, and functionalization of the carbon nanotube surface. Following the conjugation of MF to CNTs using a PEG linker, a significant improvement in the anti-neoplastic efficacy and compatibility with erythrocytes was reported (Figure 4). Pharmacokinetic investigations have shown that the presence of PEGylated CNTs leads to an increase in the retention period of MF in the biological system. This offers the prospect of decreasing the frequency of dosage for the phytochemical [37]. The results showed that CNT-PEG-MF increased bioavailability by a factor of four compared to the pure drug. It successfully induced apoptotic cell death in U-87 MG cells and showed a lengthy half-life in the body.

CNTs for bone marrow cancer detection and therapy

**Figure 5:**
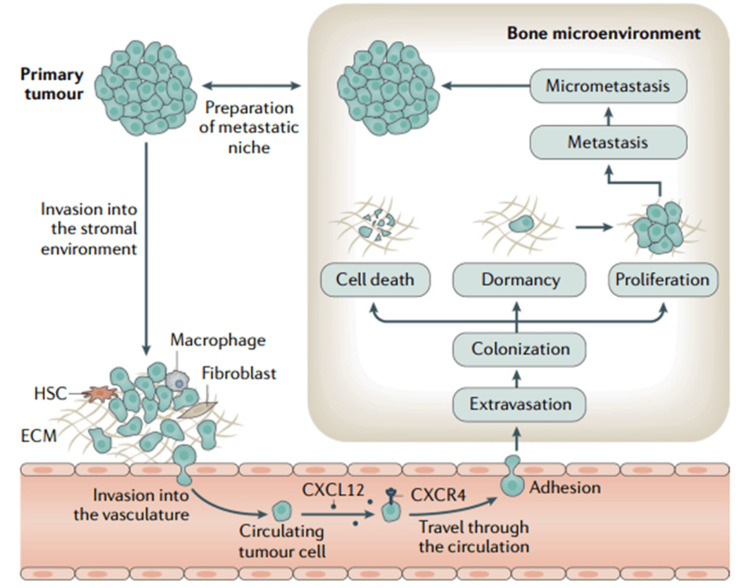
Cancer cells that have spread to the bone via metastasis. Invasion, circulation, extravasation, colonization of the metastatic location, tumor dormancy, and development to overt metastases are the phases of metastasis. In most cases, metastasis begins long before a cancer diagnosis is made. The phenomenon of tumor dormancy, however, implies that the development of overt metastases frequently does not occur until several years after the diagnosis and treatment of the initial disease. This is especially true for breast cancer and prostate cancer. Hematopoietic stem cells (HSCs), extracellular matrix (ECM), and C-X-C motif chemokine receptor ligand 12 (CXCL12) Reproduced with permission from ref. [40] Copyright 2020 Springer Nature

Bone tissue is a main destination for the spread of malignant tumors due to the presence of adhesion receptors in the endothelial cells of bone marrow and the poor blood flow in this area. Cancer cells proliferate by a series of atypical activation pathways facilitated by the niches tumor cells create in the bone marrow (Figure 5). The activation of osteoclasts, the disintegration of bone, or the enhancement of osteoclast activities may all be traced back to the production of cytokines, which is triggered by aberrant behaviors. In turn, growth factors produced by osteoclasts affect both the progression of cancer cells and bone resorption [39,40].

**Figure 6:**
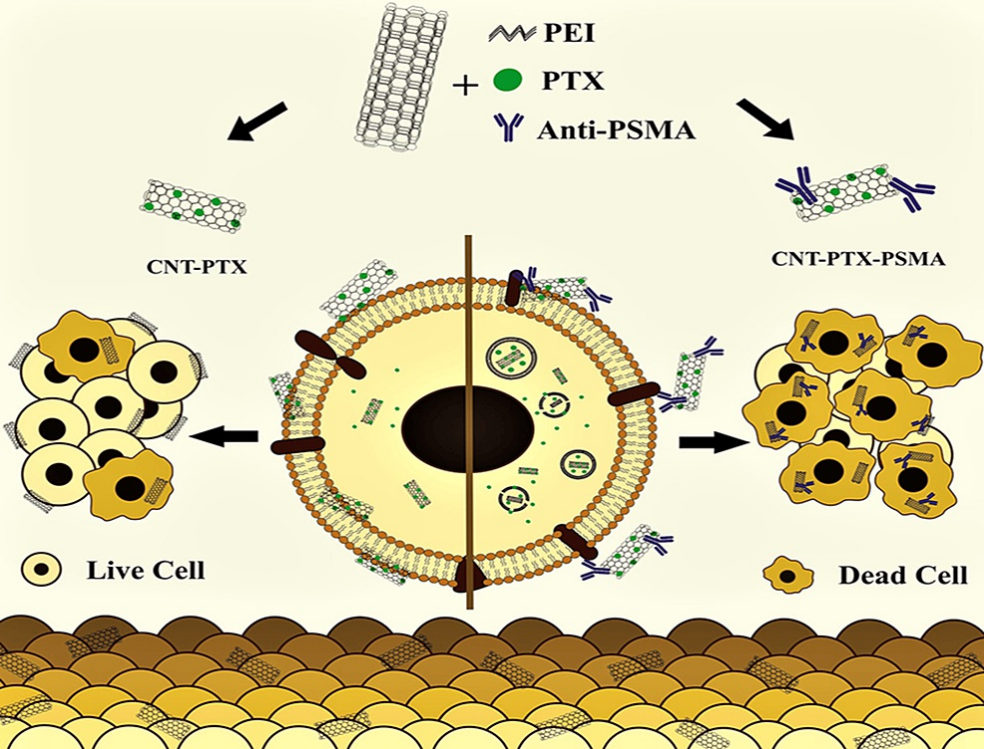
Mechanism of action of CNT -PTX on prostate cancer cell PEI: polyethyleneimine, PTX: paclitaxel, PSMA: prostate-specific membrane antigen, CNT-PTX: paclitaxel modified carbon nanotube, CNT-PTX-PSMA: paclitaxel-prostate-specific membrane antigen modified carbon nanotube. Reproduced with permission from ref. [29] Copyright 2020 Elsevier B.V.

Excessive or inadequate production of different blood cells, such as those linked with prostate, breast cancers, and lymphoblastic leukemia [41,42], indicates bone marrow abnormalities. Taghdisi et al. undertook a study to examine the use of carbon nanotubes functionalized with aptamers for delivering daunorubicin in treating acute lymphoblastic leukemia. The anticancer drug daunorubicin has been delivered to cancer cells with the help of aptamers. U266 cell concentration rose significantly, and cell viability increased to as high as 78% (p 0.005) [43]. Paclitaxel (PTX), the first-line prostate cancer treatment, was added to polyethyleneimine-functionalized multiple-wall carbon nanotubes (CNTs) by Edson José Comparetti et al. These particles were then coated with PSMA antibodies to target prostate cancer cells. HCT-116 LNCaP prostate cancer cells (PSMA+), CaCo-2 colon cancer cells (PSMA−), and human peripheral monocytes and lymphocytes (PSMA−) were in vitro exposed to fluorescent CNT composites. Fluorescence microscopy and flow cytometry showed that CNT-PTX and CNTs interact diffusely with all cell types. Cytotoxicity analysis showed that PTX complexed with CNTs was more effective on the prostate (PSMA+) and colorectal cancer cells (PSMA−) than pure PTX or CNTs alone (Figure 6) [29]. The summary of the latest studies involving CNT-based materials for cancer treatment is given in Table 1.

**Table 1:** Various carbon materials CNTs role in cancer treatment CNT: carbon nanotube, MWCNTs: multi-walled carbonanotubes, SWCNTs: single-walled carbonanotubes, DOX: Doxorubicin, ROS: reactive oxygen species, FA: folic acid, LyP-1: cryptic CendR peptide, MDA-MB-231: cell line (isolated at M D Anderson from a pleural effusion of a patient with invasive ductal carcinoma) is commonly used to model late-stage breast cancer.

Nanocomposites	Cancer type	Biomarkers	References
MultiWall Carbonanotubes-Gemcitabine	Colorectal cancer	Activates apoptosis and blocks DNA replication during S-phase.	[35]
DBCO-PEG5-NHS ester-modified CNTs	Hepatocellular carcinoma	Golgi protein 73 and Alpha-fetoprotein	[44]
Single Walled- CNTs-Capecitabine	Colorectal cancer	Type II nanocrystalline cellulose (II-NCC) interacts synergistically	[34]
Doxorubicin MWCNTs	Liver cancer	Activation of caspases inside of cells	[45]
Multi Walled- CNTs-Doxorubicin	Breast cancer	Overexpressed FA receptors should be actively targeted.	[31]
LyP-1 conjugated siRNA/MWCNTs	Pancreatic cancer	Delivery of siRNA	[46]
Functionalized CNTs	HepG2 cell line	Sorafenib delivery	[47]
SWCNTs-Doxorubicin	Breast cancer	In MDA-MB-231 cells overexpressing DOX, CD44, intracellular delivery is greatly increased.	[48]
Paclitaxel Sinwalled-CNTs	Lung cancer	Increase ROS, which activates protein kinases and destroys cancer cells.	[49]

Carbon quantum dots

The bio-imaging community has become interested in CQDs due to their novel visible-range photoluminescence capabilities [50]. This is due to their strong photo-stability, wide excitation spectrum, and adjustable emission characteristics, as well as their compact size, passivated surface, and outstanding fluorescence features [51,52]. Carbon nanomaterials might improve the efficiency of medical imaging and medication delivery [53]. Graphene quantum dots (GQDs) and Mesoporous silica nanoparticles are presented as a nanocomposite nanoparticle tool for enhancing doxorubicin administration and fluorescence imaging. As a result, researchers can monitor the drug's diffusion from the carrier as it moves across the cell [54]. New avenues of research into the identification of cancer cells may be possible if CDs are used. Cyclodextrins with added functionality may go inside many different types of cancer cells. This allows for a more precise examination of cancer cells by fluorescence imaging. Each surface group on a cancer cell and each functioning CD need a unique identification method to interact [55]. PEGylated cyclodextrins, such as those mentioned, cannot be overwritten from the user's description because of their ability to specifically target the surface moiety of angiopep 2 present in glioma cells. Because of this unique identification mechanism, imaging of gliomas is more sensitive than imaging of normal brain tissue [56]. Several ongoing studies and projects aim to create biocompatible CDs with a QY of 20% or more. Discs like this have been developed for biosensing and bioimaging applications [57].

Using passivation and doping methods on carbon quantum dots may make them more suitable for bioimaging. CDs with a high QY might store enough data to be helpful in bioimaging [58]. Incorporating heteroatoms into carbon dots, which may modify their inherent characteristics and raise the fluorescence quantum yield, may increase their potential utility in fluorescent bio-imaging. It is revealed that the quantum yield of CDs may be increased by 16% using nitrogen doping [59]. Confocal microscopy research looked at carbon dots for their luminescent properties as a possible bio-imaging probe. In this study, graphene quantum dots were made from carbon black [60]. Michigan Cancer Foundation-7 (MCF-7) human breast cancer cells uptake and produce green fluorescence following exposure to the product, suggesting its excellent biocompatibility [61,62].

Carbon Dots (CDs) have the potential to be utilized as a photosensitizer, facilitating the generation of reactive oxygen species in the presence of light [63]. The academic editing of user-provided text is impossible due to its brevity. Carbon dots may be modified in three different ways: by surface passivation, heteroatom doping, and the edge effect [64]. They developed a microwave-assisted pyrolysis method requiring only a single container to make luminous carbon dots doped with poly-dopamine. Without resorting to PDA, the insertion of N- N-atoms onto CDs tripled their quantum yield. This results in exceptional compatibility with biological systems and dependable photo-thermal conversion efficiency [65].

Targeted nano-carrier carbon quantum dots

To enhance the distribution of the pharmaceutical ingredient in the tumor while minimising exposure to healthy tissues, nano-carriers typically work by carrying medications directly to the afflicted region. This reduces the total dose of the therapy and the collateral damage to surrounding tissues [66]. Superior mechanical strength, biocompatibility, regulated API release, and simple conjugation with bioactive compounds are all desirable qualities in a drug-delivery system material. Quantum dots have been heralded as a game-changing development in the medical industry because of their unique properties. Small size, regulated drug release at the cellular level, unique surface chemistry, optical characteristics, functionalization, and photoluminescence are only a few of the advantages of nanoparticles. This has led to the use of QDs as nanocargos for drug delivery, ushering in a new age in the healthcare sector [67-69]. The chemotherapy drugs doxorubicin [70], methotrexate, cisplatin, paclitaxel [71,72], boldine, 5-fluorouracil, lisinopril [73] and, flutamide, are more able to penetrate and exert their therapeutic effects on the cancer cells. As a result of their desperation, these agents become hostile to healthy cells and cause damage to them. As a result, inadequate and potentially hazardous distribution of anticancer drugs is one of the biggest issues with chemotherapy. On top of that, drug carriers have a special quality that makes it possible to study nano-carrier activity in a living organism. Due to their ability to effectively transport medicinal medications to targeted areas while minimizing dispersion, nanoparticles have attracted much attention as part of drug delivery systems in recent years [74].

Carbon quantum dots have attracted a lot of attention as accurate and trackable carriers for targeted drug delivery due to their unique properties [75,76]. To further improve CQDs' fluorescence and quantum yield, researchers have created a number of simple production techniques, doping procedures, and precursor materials. Coating the quantum dots's surface may make it more stable, water-soluble, and target-specific while also decreasing its toxicity [77,78]. The growth of carbon quantum dots improves their solubility, biocompatibility, and cellular uptake of drugs that are otherwise insoluble [79]. Only quantum dots that fluorescence, or emit light, may be considered really advantageous. Among these benefits are biocompatibility, a novel emission mechanism, medication delivery, and superior imaging capabilities [80]. Many methods utilised to integrate therapeutic compounds into carbon quantum dots, including dissolution, adsorption, and non-covalent bonding mechanisms i.e., hydrogen bonding and electrostatic interactions.

Green-fluorescing carbon quantum dots were synthesised by researchers utilising microwaves. In hepatocellular carcinoma animal models, these carbon dots were employed to deliver drugs in a targeted fashion. More doxorubicin was coupled non-covalently with quantum dots by the researchers. Tumor stability and improved delivery efficiency were shown in vivo with the CQD-DOX combination [75]. Citric acid and o-phenylenediamine were utilized in a hydrothermal synthesis by researchers to create biocompatible fluorescent carbon quantum dots. Doxorubicin is a cationic chemotherapeutic drug, and the carbon quantum dots quickly absorbed it due to their negative surface. The fluorescence of carbon dots was turned off when they were loaded with DOX, but it was restored once the anticancer drug was washed off the surface [81]. Carbon quantum dots may be improved for bioimaging applications through passivation and doping processes. CDs with a high QY could be useful in bioimaging because of the information they could store [58]. Carbon dots' potential application in fluorescent bio-imaging could be increased by incorporating heteroatoms, which can alter their intrinsic properties and boost the fluorescence quantum yield. Nitrogen doping is found to increase the quantum yield of CDs by 16% [59]. Carbon dots were studied for their luminous qualities as a potential bio-imaging probe in confocal microscopy. Graphene quantum dots were produced from carbon black and used in this research [60]. Since the product displayed green fluorescence after being taken up by MCF-7 human breast cancer cells, we can infer that its biocompatibility is quite high [61,62].

**Figure 7:**
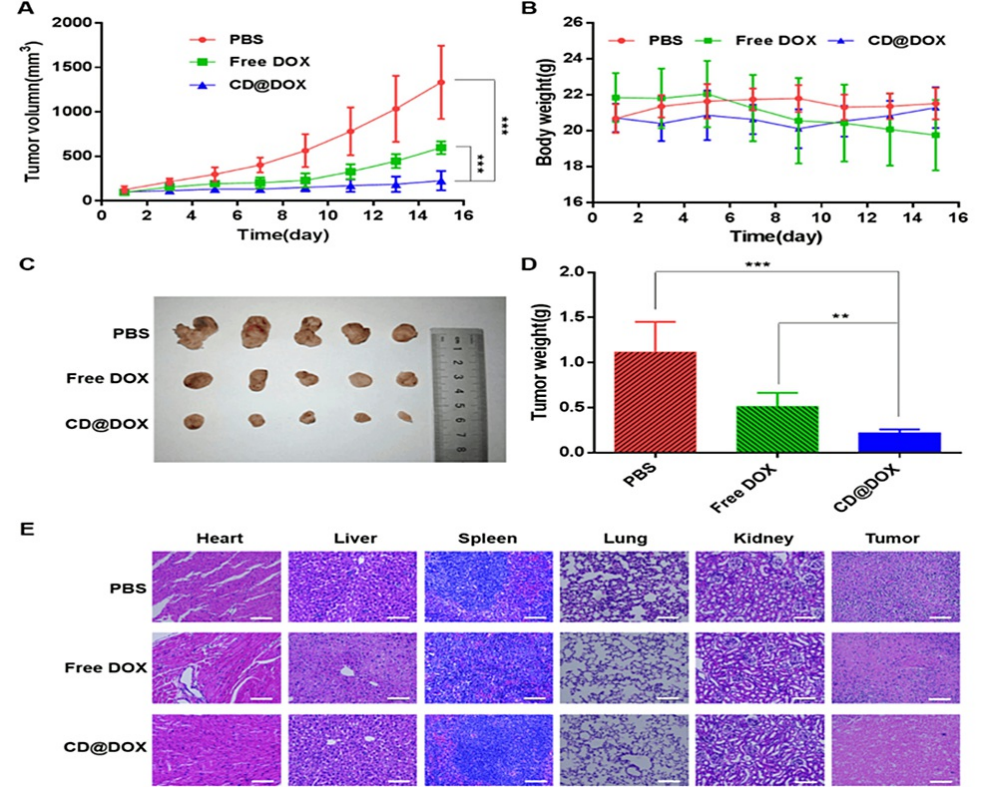
(A) The effects of injecting free doxorubicin and HA-CD@p-CBA-DOX into tumor-bearing heterotopic 4T1 mice on tumor volume. (B) The effects of free doxorubicin and HA-CD@p-CBA-DOX on the body weight of heterotopic 4T1 tumor-bearing mice. Picture (C) of the tumor. (D) Tumor masses at day 15 for all groups. (E) Tumor and major organ H&E staining after therapy. **p 0.01 and ***p 0.001 (n = 5, mean SD). The 100 m scale bar represents one hundred micrometers PBS: phosphate buffered saline, DOX: doxorubicin, CD@DOX: HA competition assay of HA-CD@p-CBA-DOX, HA: hyaluronic acid, HA-CDs: hyaluronic acid modified carbon dots, HA-CD@p-CBA-DOX: hyaluronic acid modified carbon dots loaded with doxorubicin. Reproduced with permission from ref. [82] Copyright 2020 Elsevier B.V.

CDs may be used as a photosensitizer, allowing for the easier production of reactive oxygen species when exposed to light [63]. Short user-provided content precludes scholarly rewriting. Surface passivation, heteroatom doping, and the edge effect are the three ways carbon dots can be functionalized [64]. We used a one-pot microwave-assisted pyrolysis approach to create poly-dopamine passivated fluorescent carbon dots. The quantum yield of CDs was increased by a factor of three due to the insertion of N- N-atoms, achieved without using PDA. Because of this occurrence, we now have a reliable photo-thermal conversion efficiency and unprecedented compatibility with living systems [65]. A novel drug delivery platform using hyaluronic acid-modified carbon dot-doxorubicin nanoparticles was synthesized easily. One-step hydrothermal treatment with citric acid and branch-PEI as core carbon source produced CD44-targeted HA-modified carbon dots (HA-CDs) as carriers in one hour. This method used HA as a carbon dot, hydrophilic group, and targeting ligand. The as-prepared HA-CDs were loaded with doxorubicin (HA-CD@p-CBA-DOX) through an acid-cleavable bond, which released the drug pH-responsively. In vitro tests, HA-CD@p-CBA-DOX showed excellent 4T1 cell cytotoxicity, hemocompatibility, and serum stability. Confocal laser scanning imaging and flow cytometry showed that 4T1 cells ingested DOX-loaded nanoparticles through HA-mediated CD44-targeting. Live imaging revealed that HA-CD@p-CBA-DOX increased tumor accumulation in vivo. In heterotopic and orthotopic 4T1 cell tumor models, HA-CD@p-CBA-DOX outperformed free DOX in vivo (Figure 7A, 7B). HA-CD@p-CBA-DOX's biocompatibility was further validated by blood hematological and biochemistry tests showing no harm. HA-CD@p-CBA-DOX offers a targeted breast cancer treatment approach (Figure 7C-7E) [82].

Quantum dots for tumor imaging

Tumour imaging technology is not intended for treatment but serves as a tool to inform critical choices for more effective therapy [74]. Its main function is to observe and track changes in the tumor microenvironment at the cellular, tissue scales, and subcellular. It offers comprehensive data on cancer diagnosis, screening, stage assessment, and recurrence analysis via ongoing monitoring, aiding in informed treatment choices. Additionally, they aid in comprehending the efficacy of the therapy for cohesive repairs. Various imaging techniques are used in the field of oncology; however, they encounter difficulties in terms of sensitivity and resolution, which limit their effectiveness in addressing important clinical issues such as screening, cancer staging, and therapy. Crucially, they are unable to correlate the cancer cluster with low figures.

In-vitro imaging

Researchers have been exploring the use of quantum dots for in vitro cell imaging because of the appealing properties of fluorescent carbon quantum dots, such as increased cellular uptake, the ability to preferentially target cells (via ligand conjugation), and biocompatibility. When coupled with biorecognition molecules, quantum dots also function as sensitive probes [83,84]. The quantum dots (have considerable absorption coefficients across wide spectral ranges, controllable fluorescence emission properties, high brightness, and resistance to photodegradation, all based on their size and composition. Because of this lack of steric hindrance, the binding capacities of bioconjugated quantum dots are not limited. Due to their unique properties, quantum dots have emerged as a promising tool in the field of cellular imaging. Covalent alteration of the surface of fluorescent carbon dots is often used to fine-tune their shape, size, and physical properties. The many types of functional groups on the surface may be used to add covalent changes, including amide coupling, sialylation, esterification, sulfonylation, and copolymerization. Due to electrostatic contact, complexation processes, or interactions, quantum dots can maintain structural integrity after non-covalent modifications [85]. Researchers developed graphene quantum dots that do not need dye. In addition to its other applications, such as cell imaging and drug delivery, these graphene dots were also employed to monitor the dynamic process of cellular uptake in real time [86].

In-vivo imaging

In addition to their use in in-vitro imaging, fluorescent carbon quantum dots have been the subject of extensive study in in-vivo animal models. Using a light bleaching technique, researchers created very long-lasting fluorescent carbon dots [87]. They thought the carbon quantum dots' combination of high bio-imaging quality, low cytotoxicity, and strong antioxidant properties made them ideal for medical applications. Fluorescent images are captured at several time points after intravenous administration of the carbon dots to the mouse model. Results showed that quantum dots displayed fluorescence within 5 minutes of administration and accumulated at the cancer site. After 12 hours, all of the tumor's buildup had been seen, and it was demonstrated to remain during the long imaging session. Images of animal organs after dissection showed that carbon quantum dots were successfully concentrated in tumors, kidneys, and the liver. However, no fluorescent signals were detected in vital organs, including the heart and lungs. Fluorescent signals were also not detected in the spleen [87]. Table 2 gives the summary of various carbon quantum dots nanocomposites in the treatment of cancers.

**Table 2:** Role of carbon/quantum dots against various cancers HA: hyaluronic acid, HA-CDs: hyaluronic acid modified carbon dots, DOX: doxorubicin, HA-CD@p-CBA-DOX: hyaluronic acid modified carbon dots loaded with doxorubicin, TRITC: tetramethylrhodamine-5-isothiocyanate, UCNP: upconversion nanoparticles, GQD: graphene quantum dot, UCNP-GQD/TRITC: upconversion nanoparticles-graphene quantum dot-tetramethylrhodamine-5-isothiocyanate, CQDs: carbon quantum dots, PDT: photodynamic therapy, PEG: polyethylene glycol, hMSN: hollow mesoporous silica nanoparticles, GQDs@hMSN(DOX)-PEG: graphene quantum dots incorporated into the cavity of hollow mesoporous silica nanoparticles with high cargo-loading efficiency achieved for doxorubicin, Se/N-CDs: Se/N-doped carbon dots, Lu: lutetium, TP: texaphyrin, Gd: gadolinium, RGD: arginyl-glycyl-aspartic acid, Lu-TP&Gd-TP/GQD-RGD: lutetium (III) texaphyrin-Gadolinium (III)/graphene quantum dot-arginyl-glycyl-aspartic acid, BRT: biological redox therapy, PTT: photo-thermal therapy

Nanocomposite	Binding force	Synthesis	Highlights	References
HA-CD@p-CBA-DOX	acid-cleavable bond	one-step hydrothermal	HA-CD@p-CBA-DOX showed better in vivo anti-tumor activity in heterotopic and orthotopic 4T1 cell tumors.	[82]
UCNP-GQD/TRITC	GQD-UCNP and (TRITC-UCNP-GQD) covalent bonded	Hydrothermal method	Targeted mitochondrial photodynamic treatment with near-infrared light	[88]
Carbon dots	Non-covalent electrostatic attraction, Covalently bonded and H-bonding	-	Their high quantum yield makes them an attractive bioimaging, sensing and targeted chemotherapeutic option.	[89]
CQDs	-	Nucleic acid Targeting	superior in vitro efficacy of PDT for cancer cell killing	[90]
GQDs@hMSN(DOX)-PEG	Covalent bond	Hydrothermally	PDT and an improved medication delivery system	[91]
Se/N-CDs	Electrostatic interaction	Isothermal	PDT in the nucleus made Se/N-CDs more effective in inhibiting tumor growth in vitro and in vivo.	
Lu-TP&Gd-TP/GQD-RGD	hydrophobic interactions, and π-π stacking	Hydrothermal method	PDT, BRT, and PTT are only some of the many possible combination therapies.	[92]
GQDs	controlling oxygen content using self-enriched O2	Immobilization of catalase cell membrane coating	Tumor-specific accumulation, extended circulation times, and homotypic targeting mechanisms directed towards malignant cells.	[93]
GQD-SS-Ce6	Disulfide bond	Improved Hummers method	A redox-responsive photodynamic nanosystem that effectively inhibits tumor development.	[94]
GQDs	_	Peeling and exfoliating graphite flake	The ROS production from GQDs is much higher than that from regular photosensitizers.	[95]
Cationic carbon dots	nucleus targeting	Hydrothermal approach	enhanced cytotoxicity toward malignant cells	[96]
N-GQD-DOX-APTES	Covalent bond	Hydrothermal	Drug delivery to the nucleus with variable PTT	[97]

Graphene

Several oxygen-containing functional groups on the basal plane and the edges provide GO greater hydrophilicity than graphene. Water's suitability for use in nanomedicine is much improved by the addition of these capabilities since hydrogen bonds may now be formed in it [98]. Like graphene, this nanomaterial can convert the thermal energy of near-infrared light into usable forms of heat. Hyperthermia is produced, and cancer cells are killed off by heat when this method is employed to treat the disease [99,100]. Due to its large surface area and high loading capacity, GO is a promising nanoplatform for PTT/PDT hybrid therapy. Graphene oxide-based nanocomposites containing Gadolinium ion, a contrast agent in magnetic resonance imaging, have been synthesized and reported in many publications. Graphene oxide was used to successfully create a theranostic nano-delivery system that combines anticancer medications with MRI-compatible gadolinium and gold nanoparticles. The system's effectiveness as a chemotherapeutic and diagnostic agent has been shown in vitro [101]. The use of chemotherapy, photo-thermal therapy, and photodynamic therapy in combination with theranostic systems based on graphene oxide and including compounds with cancer-targeting properties, such as folic acid or hyaluronic acid, has also been studied for the treatment of solid tumors [102,103]. The use of hybrid composites of graphene oxide and iron oxide nanoparticles for magnetic hyperthermia therapy [104]. Laboratory experiments indicated that these systems loaded with anticancer drugs were safe to use and even more effective against cancer than the drugs used alone. Magnetic nanoparticle imaging was combined with chitosan-grafted GO [105]. Doxorubicin was loaded onto this pH-sensitive nano-carrier. It was shown to be very compatible with the L929 cell line, which is generated from healthy mouse connective tissue. In addition, the efficiency of T2 contrast was enhanced when high molecular weight chitosan was grafted onto the nano-carrier. Graphene oxide nanocomposites have been developed in recent research with the goal of treating large or deeply rooted solid tumors [106-108].

Many anticancer agents, such as Doxorubicin, Paclitaxel, and methotrexate, can be easily integrated onto the surface of GO or immobilized via graphene oxide, paving the way for the development of a drug delivery system using GO with the express goal of anticancer therapy [109]. In order to efficiently target cells at lower dosages, the use of nanoparticles specific to certain cells as carriers for medicine has shown promise [110]. For instance, one study looked at the feasibility of synthesizing aptamer-coupled magnetic graphene oxide nano-carriers to target the MCF-7 human breast cancer cell line.

In the context of cancer therapy, the nano-carriers were shown to be excellent drug carriers for targeted drug delivery systems [111]. Researchers found that Graphene oxide may be effectively copolymerized with -cyclodextrin. Methotrexate and doxorubicin, two drugs with hydrophobic and hydrophilic properties, were incorporated into the polymer by copolymerization. To a large extent, the copolymer successfully induced cytotoxicity in cancer cells [112]. A separate investigation was conducted to create superparamagnetic iron oxide-reduced graphene oxide, and then the DOX medication was loaded onto the material. The pH value at proximity to the cancer cells is 4.3. Since graphene is sensitive to acidic environments, this condition is ideal for effectively releasing medicines coated on graphene oxide [113]. Graphene oxide was used effectively as a carrier in combination with hyaluronic acid as the copolymer in a single study to encapsulate the chemotherapy drugs doxorubicin and paclitaxel. Primary emphasis was placed in this study on BT-474 and MDA-MB-231 breast cancer cell lines. The results showed that apoptosis was induced by the chemical cocktail but that BT-474 cells deficient in CD44 receptor expression were resistant to the treatment [114].

**Figure 8:**
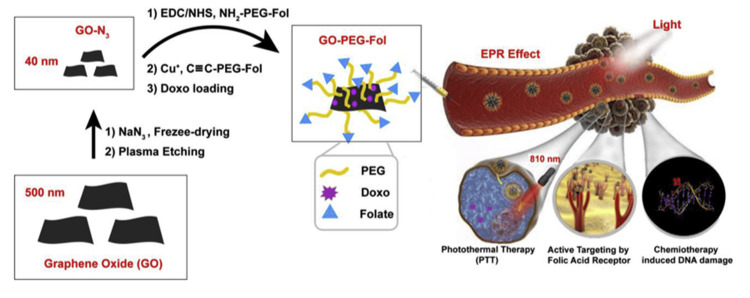
Combining NIR anticancer phototherapy with targeted medication delivery using graphene oxide nanosheets functionalized with folic acid by plasma etching Reproduced with permission from ref. [117] Copyright 2019 Elsevier B.V.

DOX-loaded PEG-PCL micelles were conjugated with avb3 integrin using this method. Cancer endothelial cells have been shown to take up the complex by studies examining the enhancement of endocytosis mediated by receptors [115,116]. Researchers set out to determine whether or not adding oridonin, an anticancer drug coated with graphene oxide, to the GE11 peptide copolymer increased its effectiveness against cancer. This research aimed to increase graphene oxide's uptake by esophageal cells with an excess of epidermal growth factor receptors. Researchers used a graphene oxide-polyethylene glycol-folic acid-conjugation approach to precisely characterize breast cancer cell lines, including MCF-7 and MDA-MB-231 (Figure 8). Through this conjugation process, we saw the incorporation of abundant DOX and the subsequent creation of features like a near-infrared light-activated heater [117].

**Figure 9:**
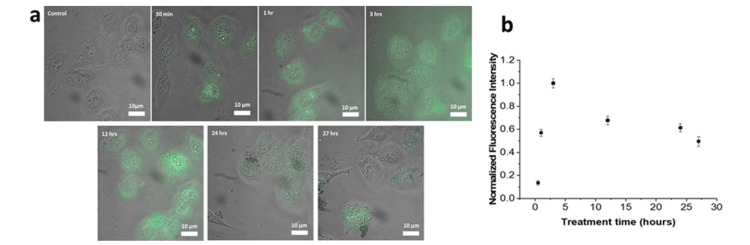
Shows (a) images of the fluorescence of GO-Fe3O4 in HeLa cells at various periods after transfection and (b) the internalization of GO over time, measured by the average normalized intensity per unit emissive area of GO-Fe3O4 fluorescence in HeLa cells Reproduced under the terms of the Creative Commons Attribution License from ref. [118] Copyright 2019 Gonzalez-Rodriguez et al. PLOS ONE

Graphene oxide and superparamagnetic iron oxide integration have the potential to improve functionality and efficiency. Non-covalently bound doxorubicin is transported favorably by fluorescence tracking in the GO-Fe3O4 composite material. Due to the high DOX loading achieved by conjugation, GO's effectiveness is increased by a significant 2.5-fold (Figure 9A, 9B) [118]. A detailed summary of various graphene-based materials is given in Table 3.

**Table 3:** Graphene-based materials for effective treatment of cancer cells GO: graphene oxide, DOX: doxorubicin DTPA: diethylene triamine penta acetic acid

Nanocomposites	Size	Cancer cell line	Drug loading efficiency	Drug Used	Drug loading efficiency	Stability		Ref
Graphene oxide/ gemcitabine/ montmorillonite/ chitosan	130 nm	MB-231-MDA cells	-	Gemcitabine	Within 24 hours, at a pH of 7.4, 23%	Excellent physical, chemical, and thermal stability. The time frame is not specified.		[119]
Graphene oxide -PEG	-	Colorectal cancer tumours	90%	5-Fu	Acidic solution (pH = 5.6), 65% of 5-FU was released in the first 12 hours and 84% throughout 72 hours.	Enhanced biocompatibility and stability		[108]
Cyclodextrin dendritic-Graphene oxide	-	Human breast cancer cells	9.8%	DOX	Within 144 hours, 67% at pH 5.2 and 85% at pH 7.4	-		[120]
Superparamagnetic graphene oxide	9.3 (±2.7) nm	MCF-7, HeLa, and Caov-4 cancer cell lines	75%	Methotrexate	pH 7.4: 46% (3.1), pH 5.5: (3.3), 59% in 75 hours	functional at physiological pH. Lack of context about stability duration		[121]
Ferric oxide/ Reduced graphene oxide/ chitosan/ doxorubicin	60 nm	MCF-7 and A549 cancer cells	98%	DOX	within 10 hours, 96.6% at pH 5.5 and 10% at pH 7.4	-		[122]
Mesoporous silica/nanoparticles/GO/ topotecan	190 nm	MDA-MB-231 cells	36.6%	Topotecan	pH 5.5: 76.0%, pH 7.4: 45.0% within 24 hours	-		[123]
cyclodextrin/cystamine/ pegylated functionalized graphene oxide	531 nm	human liver cancer cell line	95.5%	DOX	72 hours later, 65.2% at pH 7.4 and 37.6% at pH 5.3	Excellent resistance to the salt solution found in the human body. There is no set time limit.	[124]	
Amino acids-functionalized GO foams	80 nm	HepG2 and MCF-7 cell lines	67.55%	Cisplatin	Within 7 hours, 68.1% at pH 7.4	Excellent biocompatibility and storage stability.		[125]
miRNA/–polyethylene glycol/nano-GO/folic acid - platinum	200–500 nm	SKOV 3 cells, and SKOV 3 DDP cells	6%	Platinum	Proportion: 90% at pH 5, -63% at pH 7.4 Under a day	Excellent two-week stability in water, MES, PBS, and cell media.		[126]
Graphene oxide/magnetic iron oxide nanoparticles	<100 nm	Human neuroblastoma/SH-SY5Y cells	-	DOX	Within 120 hours, the proportion is 28% at pH 7.4 and 45% at pH 5.5.	increased solubility in water and stability for 24 hours.		[127]
Paclitaxel/ graphene oxide/gold nanorods loaded into poly (tetramethylene ether) glycol/polyurethane	average length and width of 34 ± 3 nm and 9.8.0 ± 1.2 nm	Human lung cancer cell lines/ A549	-	Paclitaxel	80% after 96 h and 120 h under pH values of 5.5 and 7.4, respectively	-		[128]
GO- integrated with polydopamine-bovine serum albumin	70 -180 nm	Wistar rats	~75%	DTPA-Mn(II)	During the first 5 hours of incubation at pH 7.4, 75% of was immobilised.	-		[129]

Future outlook

Although NPs have shown promise as a new treatment method, there has been little progress in incorporating them into clinical practice due to knowledge gaps around their behavior and toxicity in humans. To enable the comparison of results across different research institutions and to develop a consensus on the toxicity and pharmacokinetics of these particles, it is crucial to characterize nanoparticles in detail and execute standardized experimental techniques. To further advance NPs towards clinical use, it is critical to examine innovative targeting and biomimetic techniques, such as using NPs coated with cancer cell membranes.

Despite the above benefits, various obstacles prevent the widespread use of treatments based on carbon-based materials (such as graphene, CNTs, Carbon quantum dots, and carbon nanotubes) in clinical practice. For instance, not enough research has been done to fully understand the risks associated with using carbon-based materials within the human body. The safety of carbon-based materials has been shown via in vitro investigations on different cell lines, although in vivo testing employing animal models is normally undertaken over a short period. Further study is required to determine the long-term safety of carbon nanotube-based nanomedicine and its potential therapeutic use in the human body. In addition, many methods for altering carbon-based materials are rather complex when it comes to mass production. As a result, the difficulty in creating dependable and repeatable industrial manufacturing of functionalized carbon-based materials is a barrier to their use in therapeutic settings. When it comes to large-scale production, however, carbon quantum dots outperform other nanomaterials thanks to their simple synthesis process, low cost, and low environmental impact. Unfortunately, it has been discovered that the toxicity of CQDs increases with increasing concentration, limiting their usefulness. Therefore, it is crucial to evaluate and improve the accuracy of cancer cell targeting or targeting of TME components in carbon-based treatments to reduce toxicity. Graphene and its derivatives have outstanding electrical characteristics, making them very attractive for cancer biosensing in terms of diagnostic capacities.

## Conclusions

In the realm of cancer research, nanomaterials show great promise as promising materials for developing innovative diagnostic and therapeutic platforms. The use of metal and carbon-based nanoparticles has been shown to improve biosensors' performance in diagnostics. The rate of electron transport in electrochemical electrodes may be increased by increasing their electroactive surface area. In this setting, NPs may also act as redox mediators. Potential applications for nanoparticles include their use as colorimetric probes and incorporation into biosensors based on the phenomenon of surface plasmon resonance (SPR). It has been discovered that they possess antitumoral qualities that allow them to efficiently target oxidative stress, energy metabolism, and drug resistance, all of which are hallmarks of cancer.

Additionally, it has been shown that the use of NPs in combination with several standard chemotherapeutic medicines might possibly induce lethal effects on cancer cells. These properties offer the potential to overcome the limitations of conventional combination chemotherapy, such as poor drug solubility, pharmacokinetic fluctuation, and difficulties in ensuring accurate spatial and temporal drug distribution. This is of special significance because NP-based nano-carrier systems may be used for smart medication delivery. While chemotherapy remains the mainstay of cancer treatment, using metal nanoparticles has shown promising results in boosting the efficiency of radiation and photodynamic therapy.
